# Efficacy, Tolerability, and Acceptability of Iron Hydroxide Polymaltose Complex versus Ferrous Sulfate: A Randomized Trial in Pediatric Patients with Iron Deficiency Anemia

**DOI:** 10.1155/2011/524520

**Published:** 2011-10-31

**Authors:** Beril Yasa, Leyla Agaoglu, Emin Unuvar

**Affiliations:** Department of Pediatric Hematology/Oncology, Istanbul Medical School, University of Istanbul, Capa 34093, Istanbul, Turkey

## Abstract

Iron polymaltose complex (IPC) offers similar efficacy with superior tolerability to ferrous sulfate in adults, but randomized trials in children are rare. In a prospective, open-label, 4-month study, 103 children aged >6 months with iron deficiency anemia (IDA) were randomized to IPC once daily or ferrous sulfate twice daily, (both 5 mg iron/kg/day). Mean increases in Hb to months 1 and 4 with IPC were 1.2 ± 0.9 g/dL and 2.3 ± 1.3 g/dL, respectively, (both *P* = 0.001 versus baseline) and 1.8 ± 1.7 g/dL and 3.0 ± 2.3 g/dL with ferrous sulfate (both *P* = 0.001 versus baseline) (n.s. between groups). Gastrointestinal adverse events occurred in 26.9% and 50.9% of IPC and ferrous sulfate patients, respectively (*P* = 0.012). Mean acceptability score at month 4 was superior with IPC versus ferrous sulfate (1.63 ± 0.56 versus 2.14 ± 0.75, *P* = 0.001). Efficacy was comparable with IPC and ferrous sulfate over a four-month period in children with IDA, but IPC was associated with fewer gastrointestinal adverse events and better treatment acceptability.

## 1. Introduction

Iron deficiency (ID) affects an estimated two billion people worldwide [1] and is one of the most common nutrient deficiencies in all regions, including Europe [2, 3]. If no corrective action is taken, ID can manifest as iron deficiency anemia (IDA), which has been linked to fatigue, weakened immunity, poor work performance, and a decreased quality of life [4]. Moreover, infants with IDA have been shown to achieve lower scores on mental and motor development tests than infants with normal iron status [5]. Inadequate intestinal absorption of nutritional iron to meet physiological requirements may occur due to inadequate iron intake, increased iron requirement (e.g., during periods of rapid growth), or chronic blood loss. Infants, preschool children, and adolescents are among the groups most susceptible to development of IDA [6].

Providing adequate iron supplementation, ideally before the development of anemia, can prevent the systemic neurological and developmental disorders that result from IDA in infancy and childhood [5]. Long-term oral iron is frequently used as a first-line therapy, but iron salts such as ferrous sulfate are associated with a high incidence of gastrointestinal side effects such as nausea, vomiting, constipation, and diarrhea [7]. Polynuclear preparations based on the ferric form of iron, such as iron hydroxide polymaltose complex (IPC), have been developed to improve tolerability. IPC provides similar iron bioavailability to ferrous sulfate [8] but has a stable structure that confers more controlled absorption of iron [9]. A recent meta-analysis [10] has confirmed that IPC and ferrous sulfate provide similar improvements in hemoglobin (Hb) levels in adult patients with iron deficiency anemia, but with superior tolerability. The available data comparing IPC versus ferrous sulfate in children suggest that efficacy is similar with the two preparations [10], but randomized trials are more rare than in adults and long-term data are lacking. Two randomized studies, one in 30 iron deficient children with or without anemia aged 24–81 months [11] and the other in 49 children with IDA aged 6–40 months [12], reported no difference between IPC and ferrous sulfate for the improvement in Hb or other efficacy markers over a two-month period, although one trial observed a more rapid improvement in Hb with ferrous sulfate. Tolerability was superior with IPC in both studies [11, 12]. 

The current study evaluated the efficacy, tolerability, and acceptability of IPC and ferrous sulfate in a cohort of 103 pediatric patients with IDA during a four-month treatment period.

## 2. Methods

### 2.1. Study Design

This was a prospective, randomized, open-label, four-month study undertaken in children with IDA at the Department of Pediatric Health and Diseases Outpatient Clinics of the University of Istanbul during 2009. Patients were eligible for enrollment if they were older than six months of age and presented with at least one of the symptoms of fatigue, faintness, or getting tired quickly, without known underlying chronic disease. Diagnosis of IDA was based on age-dependent lower limits of normal for Hb and iron status parameters (Table 1) [13, 14]. Patients with Hb values below normal were tested for transferrin saturation (TSAT), serum iron, and serum ferritin levels. If any of these iron parameters were below normal, the patient was included in the trial and randomized to iron treatment with ferrous sulfate (twice daily; Ferro Sanol Syrup, Adeka, Turkey) or IPC (once daily; Ferrum Hausmann Syrup, Abdi Ibrahim, Turkey) at a total dose of 5 mg iron/kg/day. Randomization was performed by alternating treatment allocation of newly recruited patients on a weekly basis, that is, patients who were recruited during one week were allocated to one treatment group and those recruited during the following week to the other treatment group.

The study was conducted in accordance with the Declaration of Helsinki and Good Clinical Practice guidelines. The study protocol was approved by the local Ethics Committee (registration number 2009/1897) and legal representatives of the children provided informed consent before enrollment in the study. 

### 2.2. Laboratory Evaluation

Baseline measurements comprised the erythrocyte-related hematologic markers Hb, hematocrit (Hct), mean corpuscular volume (MCV), mean corpuscular hemoglobin (MCH), mean corpuscular hemoglobin concentration (MCHC), and red blood cell (RBC) count as well as the iron status markers serum iron, serum iron binding capacity (SIBC), TSAT, and serum ferritin. Outcome assessments comprised the percentage of reticulocytes at day 7, erythrocyte markers at months 1 and 4, and iron status markers at month 4. Erythrocyte markers and reticulocytes were measured with an ABX Pentra DX 120 Analyzer. Iron parameters were assessed with standard laboratory methods using COBAS INTEGRA 800 and COBAS E autoanalyzers.

### 2.3. Tolerability and Acceptability

Gastrointestinal adverse events with a possible relation to study medication (e.g., nausea, abdominal pain, diarrhea, and constipation) that developed during the course of treatment and were reported at any study visit. Treatment acceptability was assessed using the Wong-Baker scale, which scores facial expressions on a scale of 0–5 points that reflect difficulties during administration of iron treatment [15]. A “happy face” (0) stands for no difficulties, while a “sad face” (5) indicates that the child refused or was forced to take the medicine.

### 2.4. Statistical Analyses

Data are presented as mean ± standard deviation (SD) unless otherwise stated. Statistical analyses were performed with the NCSD (Number Cruncher Statistical System) 2007 and PASD 2008 Statistical Software (Utah, USA). Student's *t*-test was used for group comparison of parameters with normal distribution. Mann-Whitney *U* test was used for group comparison of parameters that did not show normal distribution. Variance analysis was used for the detection of difference between repeated measurements of parameters showing normal distribution. Wilcoxon test was used for repeated measure analysis of parameters that did not show normal distribution. Significance was considered at the level of *P* < 0.05. 

## 3. Results

### 3.1. Patient Population

One hundred and three children were screened for eligibility, all of whom met the criteria for inclusion and were recruited to the study (42 girls, 61 boys; mean age 6.4 ± 5.1 years, range 7 months to 17 years). The patients were evenly distributed between the two treatment groups (IPC, *n* = 52, 49.5%; ferrous sulfate, *n* = 51, 50.5%). Baseline characteristics were comparable between both groups except for serum ferritin levels, which were significantly higher in patients randomized to IPC compared to ferrous sulfate (Table 2). However, baseline ferritin levels were below the age-dependent lower limit of normal in both groups.

### 3.2. Efficacy

The percentage of reticulocytes at day 7 was similar with IPC (1.41 ± 1.31%) and ferrous sulfate (1.57 ± 1.29%; *P* = 0.905). All erythrocyte-related hematologic parameters at months 1 and 4 and all iron parameters at month 4 showed a significant improvement from baseline with both treatments (Table 2). A significant improvement in Hb was observed by month 1 in the IPC group (9.5 ± 1.1 g/dL to 10.6 ± 1.0 g/dL, *P* = 0.001) and the ferrous sulfate group (9.4 ± 1.6 g/dL to 11.2 ± 0.9 g/dL, *P* = 0.001), with an increase of more than 2 g/dL in both treatment arms by month 4 (IPC 11.7 ± 0.8 g/dL, ferrous sulfate 12.4 ± 1.0 g/dL; both *P* = 0.001 versus baseline). The changes in Hb and Hct levels from baseline to months 1 and 4 were not significantly different between treatment groups, although at month 1 there was a nonsignificant trend to a greater increase in Hb in the ferrous sulfate group (IPC 1.2 ± 0.9 g/dL versus 1.8 ± 1.7 g/dL, *P* = 0.060). In terms of iron status parameters, TSAT improved from approximately 5% in each group at baseline to >20% at month 4 (IPC 5.1 ± 3.3% to 20.2 ± 15.5%, *P* = 0.001; ferrous sulfate 5.4 ± 3.5% to 22.4 ± 13.2%, *P* = 0.001) with no significant difference between the groups. The increase in serum ferritin level from baseline to month 4 was almost twofold lower in the IPC group versus ferrous sulfate (22.7 ± 26.1 *μ*g/mL versus 42.5 ± 62.0 *μ*g/mL, *P* = 0.001). 

### 3.3. Tolerability

Overall, 38.8% patients (40/103) reported one or more gastrointestinal adverse event typical for oral iron supplementation, with a significantly lower frequency of events in the IPC group (26.9% [14/52]) compared to the ferrous sulfate group (50.9% [26/51], *P* = 0.012) (Table 3). The frequencies of nausea/abdominal pain and of constipation were comparable between groups, but 25.4% of patients receiving ferrous sulfate experienced both types of adverse events compared to 1.9% of IPC-treated patients. No cases of diarrhea were reported during the study period.

### 3.4. Acceptability

**Figure 1:**
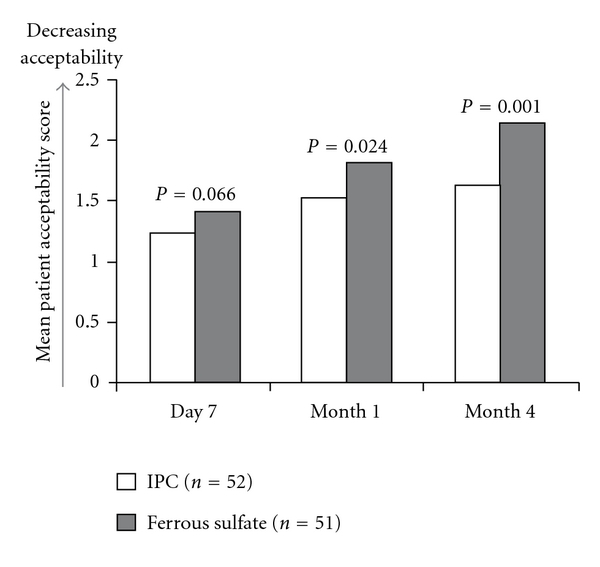
Acceptability score in pediatric patients with iron deficiency anemia receiving oral iron therapy in the form of iron hydroxide polymaltose complex (IPC) or ferrous sulfate. Acceptability was assessed using the Wong-Baker scale [15], on which a “happy face” (score 0) stands for no difficulties, while a “sad face” (score 5) indicates that the child refused or was forced to take the medicine.

Treatment acceptability at day 7 was comparable for both groups, but at months 1 and 4, the children found it significantly easier to accept IPC administration than ferrous sulfate (Figure 1). At the end of the four-month study period, the mean facial expression score on the five-point Wong-Baker scale was 0.51 points lower in the IPC group compared to the ferrous sulfate group (1.63 ± 0.56 versus 2.14 ± 0.75, *P* = 0.001).

## 4. Discussion

Results from this large, randomized study show that improvements in hematologic parameters and the availability of iron for erythropoiesis are comparable with IPC and ferrous sulfate over a four-month period in children with IDA but are achieved with fewer adverse events and improved acceptability using IPC. 

A number of studies have previously demonstrated that IPC achieves a significant increase in Hb levels in children with IDA [11, 12, 16–18]. The rate of the erythropoietic response to IPC appears to be dose dependent. In a study of 63 adults with IDA, the mean time to achieve target Hb level was 6.6, 8.3, and 11.3 weeks, respectively, for patients receiving 200, 400, or 600 mg iron [19]. At a dose of 200 mg iron/day, Langstaff et al. observed Hb increases to be higher with ferrous sulfate than IPC at weeks 3 and 6, but not at week 9 [20]. Similarly, Murahovschi et al. found in a randomized trial of 49 IDA infants that patients treated with ferrous sulfate showed a faster increase in Hb during the first month of treatment compared to those given IPC at a dose of 4 mg/kg/day, but that the increase was then slower with ferrous sulfate [12]. This may explain the lack of response to IPC 100–300 mg/day after one month described in a small study of 16 iron-depleted adults [21]. It has been suggested that the bioavailability of iron may be lower to IPC than iron salts [21, 22], but evaluation of iron bioavailability from orally administered compounds is complex, and conventional pharmacokinetic measurements of serum iron concentration are largely irrelevant in this setting [23, 24]. The true measurement of iron bioavailability is uptake of iron into the erythrocytes, which peaks at 2-3 weeks after the start of oral iron administration, and which is similar with IPC and ferrous salts including ferrous sulfate [9, 25]. In our population, the equivalent reticulocyte response at day 7 suggests that IPC rapidly provides adequate iron bioavailability for effective erythropoiesis. By month 1, there was a significant increase in Hb in the IPC cohort compared to baseline and no significant difference was seen between the IPC and ferrous sulfate arm. Of the other five hematologic parameters that were measured, only MCV showed a significantly greater improvement in the ferrous sulfate arm at month 1. At month 4, although absolute Hb was lower in the IPC arm, the change in Hb from baseline was similar between treatment groups. While levels of the storage iron ferritin were higher with ferrous sulfate, TSAT exceeded 20% in both groups, indicating that adequate iron was available for erythropoiesis. 

A drawback of oral iron supplementation, particularly ferrous sulfate, is the high incidence of gastrointestinal adverse events such as nausea, vomiting, abdominal cramps, constipation and diarrhea, and tooth staining [7, 26]. Randomized studies in adults have confirmed a lower rate of gastrointestinal symptoms with IPC versus ferrous sulfate [8, 20, 27, 28]. In children, comparative data are more sparse, but there are reports of fewer gastrointestinal adverse events [12] and less frequent tooth staining [11] in IPC-treated children compared to those given ferrous sulfate. The differences in safety profiles between the two preparations are attributed to a slower release of iron from the stable IPC complex [9]. Rapid iron release from ferrous sulfate within the gastric lumen can overload the active, control uptake mechanism in the enterocytes, leading to local gut reactions and symptoms such as vomiting and dyspepsia. Overload of the active uptake mechanism also leads to passive absorption via the intercellular route and absorption of iron from the gut directly into the bloodstream [29], with a consequent increase in nontransferrin bound iron (NTBI). NTBI iron is known to induce oxidative stress that can cause systemic adverse events including nausea. The rise in NTBI thus is negligible after IPC dosing since the size of the hydroxide complex means that there is almost no passive diffusion and the slow release of iron avoids overload of the active transport mechanism [9], but when iron is given in the form of ferrous salts, rapid release of iron means that there is a dose-dependent passive absorption of iron [29]. As a consequence, ferrous sulfate is associated with increased levels of NTBI and increased oxidative stress [28–30], whereas IPC administration is not [28, 29]. The significantly lower rate of gastrointestinal adverse events seen with IPC compared to ferrous sulfate in the current study is in line with earlier clinical experience in children [12] and with the difference in iron absorption patterns. Taking ferrous salts at mealtimes improves gastrointestinal tolerance, but markedly reduces iron bioavailability such that it is recommended to take ferrous sulfate between meals. IPC, in contrast, can be taken at meal times without compromising bioavailability [31] or effectiveness [18]. The good tolerability of IPC was confirmed in a randomized trial of IPC versus ferrous gluconate in a series of 105 healthy infants to assess their efficacy in the prevention of anemia [32]. Adverse effects such as vomiting, diarrhea, constipation, and discolored teeth were significantly less frequent in the IPC treatment group, although mean Hb levels were higher in the ferrous gluconate arm. 

The progressive increase in erythrocyte-related hematologic parameters between months 1 and 4 confirms the benefit of a long-term treatment schedule in patients given oral iron supplementation. Compliance is inevitably an issue for any long-term treatment regimen, but the high rate of gastrointestinal adverse events in infants and children given ferrous sulfate [12, 33] is likely to be an additional barrier. Limited data from studies in children and infants have suggested that compliance [34] and adherence [35] with a ferrous sulfate regimen may be as low as 30–40% over a one-week period. Two randomized studies in pregnant women have shown significantly higher compliance with IPC than ferrous sulfate [36, 37], but comparative data are not available in children. Our evaluation of the acceptability of IPC versus ferrous sulfate showed a progressive increase in the unfavorable attitude of the infants and children to ferrous sulfate compared to IPC over the four-month study period, which would tend to discourage compliance. 

In conclusion, the results of this study show that IPC is as effective as ferrous sulfate when used as an oral iron replacement therapy in pediatric patients with iron deficiency anemia. The superior tolerability of IPC compared to ferrous sulfate translated into better treatment acceptability in this population of infants and children.

## Figures and Tables

**Table 1:** Age-dependent lower limits of normal for hemoglobin and iron status parameters [10, 36].

Age (years)	Hb (g/dL)	Age (years)	Serum iron (*μ*g/dL)	TSAT (%)	Age (years)	Serum ferritin (*μ*g/L)
0.5–6	10.5	0.5–2	16	6	0.5–15	7
7–12	11.0	2–6	20	7	—	—
>12 female	12.0	6–12	23	7	>15 female	12
>12 male	14.0	>12	48	18	>15 male	15

Hb: hemoglobin; TSAT: transferrin saturation.

**Table 2:** Baseline values and changes in erythrocyte-related hematologic and iron status parameters in pediatric patients with iron deficiency anemia receiving oral iron therapy with iron hydroxide polymaltose complex (IPC) or ferrous sulfate.

	IPC **(** *n* = 52**)**			Ferrous sulfate **(** *n* = 51**)**			*P* value IPC versus ferrous sulfate	
	Value	Change from baseline	*P* value^a^	Value	Change from baseline	*P* value^a^	Absolute value	Change from baseline
Hb (g/dL)								
Baseline	9.5 ± 1.10	—	—	9.4 ± 1.6	—	—	0.849	—
1 month	10.6 ± 1.0	1.2 ± 0.9	0.001	11.2 ± 0.9	1.8 ± 1.7	0.001	0.002	0.060
4 months	11.7 ± 0.8	2.3 ± 1.3	0.001	12.4 ± 1.0	3.0 ± 2.3	0.001	0.001	0.349
Hct (%)								
Baseline	29.4 ± 2.7	—	—	29.5 ± 4.2	—	—	0.954	—
1 month	32.7 ± 2.2	3.2 ± 2.3	0.001	34.4 ± 2.8	4.9 ± 4.8	0.001	0.001	0.191
4 months	35.5 ± 2.5	6.0 ± 3.3	0.001	37.5 ± 3.2	8.0 ± 6.1	0.001	0.001	0.311
MCV (fL)								
Baseline	68.7 ± 7.9	—	—	68.4 ± 8.0	—	—	0.835	—
1 month	71.7 ± 6.8	3.0 ± 3.2	0.001	74.0 ± 5.8	5.7 ± 6.5	0.001	0.061	0.012
4 months	76.3 ± 5.3	7.5 ± 6.7	0.001	79.5 ± 5.8	11.1 ± 8.2	0.001	0.004	0.013
MCH (pg)								
Baseline	22.3 ± 3.6	—	—	21.9 ± 3.7	—	—	0.565	—
1 month	23.4 ± 3.1	1.1 ± 1.5	0.001	24.0 ± 2.7	2.1 ± 2.9	0.001	0.276	0.050
4 months	25.2 ± 2.3	2.8 ± 2.7	0.001	26.1 ± 2.6	4.2 ± 3.7	0.001	0.050	0.050
MCHC (%)								
Baseline	31.7 ± 1.7	—	—	31.4 ± 2.2	—	—	0.467	—
1 month	32.5 ± 1.4	0.8 ± 1.0	0.001	32.3 ± 1.6	0.9 ± 1.4	0.001	0.446	0.611
4 months	33.5 ± 0.8	1.7 ± 1.4	0.001	33.2 ± 1.2	1.7 ± 1.8	0.001	0.129	0.731
RBC count (×10^12^/L)								
Baseline	4.3 ± 0.4	—	—	4.2 ± 0.5	—	—	0.705	—
1 month	4.5 ± 0.4	0.2 ± 0.3	0.001	4.6 ± 0.4	0.3 ± 0.4	0.001	0.551	0.675
4 months	4.8 ± 0.4	0.5 ± 0.3	0.001	4.8 ± 0.4	0.6 ± 0.5	0.001	0.550	0.882
TSAT (%)								
Baseline	5.1 ± 3.3	—	—	5.4 ± 3.5	—	—	0.864	—
4 months	20.2 ± 15.5	15.2 ± 14.9	0.001	22.4 ± 13.2	17.2 ± 13.3	0.001	0.140	0.284
Serum ferritin (*μ*g/L)								
Baseline	10.7 ± 8.5	—	—	7.8 ± 7.6	—	—	0.007	—
4 months	33.4 ± 31.6	22.7 ± 26.1	0.001	50.3 ± 67.3	42.5 ± 62.0	0.001	0.006	0.001
Serum iron (*μ*g/dL)								
Baseline	22.2 ± 13.4	—	—	23.2 ± 13.9	—	—	0.746	—
4 months	76.3 ± 60.5	54.2 ± 58.2	0.001	75.7 ± 36.8	52.5 ± 37.7	0.001	0.210	0.432
SIBC (*μ*g/dL)								
Baseline	452 ± 68	—	—	447 ± 78	—	—	0.710	—
4 months	379 ± 46	−73 ± 54	0.001	354 ± 52	−93 ± 77	0.001	0.011	0.259

^a^ *P* value for change from baseline.

Data shown as mean ± SD.

Hb: hemoglobin; Hct: hematocrit; IPC: iron hydroxide polymaltose complex; MCH: mean corpuscular hemoglobin; MCV: mean corpuscular volume; MCHC: mean corpuscular hemoglobin concentration; RBC: red blood cell; SIBC: serum iron binding capacity; TSAT: transferrin saturation.

**Table 3:** Adverse events in pediatric patients with iron deficiency anemia receiving oral iron therapy with iron hydroxide polymaltose complex (IPC) or ferrous sulfate, *n* (%).

	IPC	Ferrous sulfate	*P* value
	(*n* = 52)		
Nausea or abdominal pain	9 (17.3)	9 (17.6)	—
Constipation	4 (7.6)	4 (7.8)	—
Nausea or abdominal pain plus constipation	1 (1.9)	13 (25.4)	—

Total	14 (26.9)	26 (50.9)	0.012
